# Hypertension, Atrial Fibrillation, and Orthostatic Hypotension in Older Adults: Blood Pressure Assessment and Treatment Decisions—A Narrative Review

**DOI:** 10.3390/medicina62101886

**Published:** 2026-09-29

**Authors:** Amela Kabaklić, Ana Spirkoska Mangaroska

**Affiliations:** 1 Department of Hypertension, Division of Internal Medicine, Dr Peter Držaj Hospital, University Medical Centre Ljubljana, 1000 Ljubljana, Slovenia; 2 Department of Internal Medicine, Faculty of Medicine, University of Ljubljana, 1000 Ljubljana, Slovenia; 3 Department of Vascular Disease, Division of Internal Medicine, University Medical Centre Ljubljana, 1000 Ljubljana, Slovenia

**Keywords:** hypertension, atrial fibrillation, orthostatic hypotension, blood pressure measurement, blood pressure variability, older adults, frailty, anticoagulation

## Abstract

*Background and Objectives:* Hypertension, atrial fibrillation (AF), and orthostatic hypotension (OH) are common in older adults and may coexist. Their coexistence challenges blood pressure (BP) assessment and treatment decisions because AF reduces measurement reproducibility, OH introduces posture-dependent BP changes, and therapies for hypertension or AF may affect orthostatic tolerance. This review examines evidence relevant to BP assessment and treatment decisions in this setting. *Materials and Methods:* A focused narrative review was conducted using targeted PubMed searches, supplemented by contemporary cardiovascular guidelines, scientific statements, consensus documents, systematic reviews, meta-analyses, and original studies. *Results:* Hypertension frequently coexists with OH in older adults, while AF adds marked beat-to-beat BP variability and reduces the reproducibility of BP readings. Orthostatic assessment identifies clinically relevant BP falls not captured by seated BP alone. Across hypertension trials, baseline OH did not appear to diminish the cardiovascular benefit of BP lowering, although frail older adults and patients with symptomatic, severe, or neurogenic OH were underrepresented. Effects on orthostatic tolerance differ by drug class and mechanism. β-blockers may blunt compensatory heart-rate responses but may still be required for AF rate control. Frailty and falls influence treatment tolerance and bleeding risk, while the fall risk alone does not generally justify withholding indicated anticoagulation. *Conclusions:* In older adults with hypertension, AF, and OH, treatment decisions should be guided by BP assessment that captures rhythm-related measurement variability and postural BP change. OH should prompt reassessment of the BP profile, symptoms, and contributing medications rather than automatic antihypertensive deintensification. Medication review should distinguish reversible contributors to orthostatic intolerance from therapies with compelling cardiovascular indications, while frailty, falls, and anticoagulation should inform risk–benefit assessment. Prospective studies are needed to establish how BP and hypertension can be reliably assessed in older adults with coexisting AF and OH and how such assessment should guide treatment.

## 1. Introduction

Atrial fibrillation (AF) is the most common sustained cardiac arrhythmia and predominantly affects older adults, with approximately 80% of affected adults aged ≥65 years [1,2]. Hypertension frequently coexists with AF and is the leading modifiable risk factor for its development [3]. Hypertension may promote arrhythmogenesis through haemodynamic and neuroendocrine mechanisms and through structural remodelling of the atria and ventricles, including myocardial fibrosis and left ventricular hypertrophy [3,4]. In older adults, AF commonly occurs in a broader context of frailty and multimorbidity, often accompanied by polypharmacy, which adds to treatment burden and complicates clinical management [2].

In AF, continuous variation in ventricular filling, stroke volume, and contractility contributes to marked beat-to-beat blood pressure (BP) variability. This variability may complicate the interpretation of individual BP readings and the assessment of BP control, particularly when only a limited number of measurements are obtained [5]. Beyond this immediate beat-to-beat variation, longer-term BP variability has also been associated with adverse clinical outcomes in patients with AF [6,7]. Reliable BP assessment in AF therefore requires repeated measurements rather than reliance on isolated readings [5].

Orthostatic BP assessment is particularly relevant in older adults with coexisting hypertension and AF. Orthostatic hypotension (OH) is common in older adults and frequently coexists with hypertension [8,9]. In older adults, OH is often multifactorial, with impaired autonomic responses, volume depletion, and medication effects among the contributing factors [8,9]. On standing, maintenance of BP depends on coordinated compensatory responses, particularly sympathetic vasoconstriction and an increase in heart rate [9]. The heart-rate response therefore provides additional information when interpreting an orthostatic BP fall, although its diagnostic value is limited in AF and during β-blocker therapy because both affect heart-rate control [8]. Observational studies have also documented an association between AF and OH [10,11]. The coexistence of hypertension, AF, and OH may complicate therapeutic decisions in older adults. Certain antihypertensive and rate-control therapies can affect orthostatic tolerance through different mechanisms. For example, diuretic-related volume depletion may contribute to OH, whereas β-blockers used for rate control may blunt compensatory heart-rate responses [9,12]. Frailty and multimorbidity may further reduce treatment tolerance and complicate therapeutic decision-making in older patients [2,12]. OH is associated with falls, while fall-related trauma increases the risk of bleeding in patients receiving anticoagulant therapy for stroke prevention [12,13]. Uncontrolled hypertension is also a modifiable bleeding risk factor in anticoagulated patients [1,13]. Balancing cardiovascular risk reduction against symptom burden, treatment tolerance, fall risk, and bleeding risk may therefore be particularly challenging in older adults with coexisting hypertension, AF, and OH. Clinical decisions in this setting should be grounded in reliable BP measurement and careful interpretation of BP changes within the patient’s broader clinical context, rather than in isolated BP values or diagnostic thresholds alone.

Evidence relevant to older adults with coexisting hypertension, AF, and OH is largely derived from separate bodies of literature on BP measurement in AF, orthostatic BP responses in hypertension, treatment-related effects on orthostatic tolerance, and the implications of frailty, falls, and anticoagulation for clinical decision-making. This narrative review critically integrates these evidence streams around two closely linked clinical questions: how BP should be measured and interpreted, and how treatment decisions should be approached when these conditions coexist in older adults. Particular attention is given to limitations of the available evidence, its generalizability to frail and multimorbid older patients, and priorities for future research.

## 2. Review Approach

This narrative review addressed two clinical questions: how blood pressure (BP) should be measured and interpreted in older adults with hypertension, atrial fibrillation (AF), and orthostatic hypotension (OH), and how these findings should inform treatment decisions. PubMed was searched for publications from 1 January 2015 to 31 July 2026 using six focused search strategies. The searches used free-text terms restricted to the Title and/or Title/Abstract fields; no MeSH terms were used. The searches were: (1) (“atrial fibrillation”[Title/Abstract]) AND (“blood pressure measurement”[Title/Abstract] OR sphygmomanomet*[Title/Abstract]) AND (accuracy[Title/Abstract] OR validation[Title/Abstract] OR reproducibility[Title/Abstract]) (38 records); (2) (“orthostatic hypotension”[Title]) AND (hypertension[Title] OR antihypertensive*[Title]) AND (treatment[Title/Abstract] OR discontinu*[Title/Abstract] OR withdrawal[Title/Abstract] OR intensif*[Title/Abstract] OR “drug class”[Title/Abstract]) (42 records); (3) (“atrial fibrillation”[Title/Abstract]) AND (“orthostatic hypotension”[Title/Abstract]) (56 records); (4) (“atrial fibrillation”[Title]) AND (frailty[Title] OR frail*[Title]) AND (prevalence[Title] OR outcome*[Title] OR anticoagula*[Title] OR bleeding[Title]) (118 records); (5) (“atrial fibrillation”[Title/Abstract]) AND (fall*[Title]) AND (outcome*[Title/Abstract] OR bleed*[Title/Abstract] OR anticoagulat*[Title/Abstract] OR stroke[Title/Abstract]) (73 records); and (6) (“atrial fibrillation”[Title/Abstract]) AND (“blood pressure variability”[Title/Abstract] OR “visit-to-visit blood pressure”[Title/Abstract] OR “systolic blood pressure variability”[Title/Abstract]) (55 records). The publication-date restriction was applied to all searches; no language, age, human-study, or publication-type filters were applied at the database-search stage. Across the six searches, 382 records were retrieved. After removal of duplicates, 375 unique records were screened by Titles and Abstracts. Reference lists of systematic reviews, meta-analyses, narrative reviews, guidelines, scientific statements, consensus documents, and original studies were screened manually.

Older adults were defined primarily as individuals aged ≥65 years. Because studies specifically enrolling older adults with coexisting hypertension, AF, and OH were scarce, studies of broader or partially overlapping adult populations were eligible when they directly informed one of the two clinical questions and evidence specific to older adults was limited. AF-specific studies that did not require coexisting hypertension and OH were included when they directly informed BP measurement accuracy and reproducibility, BP variability, or treatment-related decision-making. English-language human publications were considered during screening. Guidelines, scientific statements, consensus documents, reviews, and clinically relevant original studies were eligible. Case reports, study protocols, editorials, comments, corrections, non-human studies, and publications without direct relevance to the review questions were excluded. Differences in OH diagnostic thresholds, measurement timing, and supine-to-standing versus seated-to-standing protocols were taken into account when interpreting the evidence rather than being treated as equivalent.

Initial screening and study selection were performed by A.K. Potentially eligible or uncertain publications were discussed with A.S.M., and final inclusion decisions were agreed by both authors. Of the 375 unique PubMed records screened by Titles and Abstracts, 71 publications were assessed in full text. Of these, 21 were retained from the focused PubMed searches, and 50 were not retained. The principal reasons for non-retention after full-text assessment were limited direct relevance to the review questions, substantial overlap with more comprehensive or more directly applicable evidence, and limited applicability to the target older-adult population or to routine BP/OH assessment and treatment decisions. Supplementary literature identification, principally through reference-list screening of key publications, contributed a further 27 publications, resulting in 48 publications in the final narrative synthesis.

No formal risk-of-bias assessment or quantitative evidence synthesis was performed, consistent with the focused narrative design of the review. Methodological limitations and applicability to older, frail, and multimorbid populations were considered explicitly during interpretation.

Key studies directly informing the two clinical questions addressed in this review are summarized in Table 1, with particular attention to study design, population, BP and OH assessment, treatment or exposure, major findings, methodological limitations, and applicability to older adults.

## 3. Clinical Overlap of Hypertension, Atrial Fibrillation, and Orthostatic Hypotension in Older Adults

Hypertension and atrial fibrillation frequently coexist, and both become increasingly prevalent with advancing age. Hypertension affects approximately 60% of people older than 60 years and 75% of those older than 75 years [24]. Hypertension is estimated to be present in more than 70% of patients with AF, and individuals with hypertension have a higher likelihood of AF than those without hypertension (pooled odds ratio (OR) 1.73, 95% confidence interval (CI) 1.31–2.28) [3]. Several haemodynamic, neuroendocrine, structural, and electrophysiological mechanisms may link hypertension to AF [3,4].

Orthostatic hypotension also becomes more prevalent with advancing age, affecting an estimated 16–30% of adults older than 65 years [9]. In older adults, OH frequently coexists with hypertension [9,25]. Ageing weakens baroreflex-mediated BP regulation, and this impairment may be accentuated by hypertension and arterial stiffening. Volume depletion, medication effects, and comorbidities provide additional contributors to OH [9,23].

An association between atrial fibrillation and OH has also been documented. In the Irish Longitudinal Study on Ageing (TILDA) cohort, participants with AF were more likely to have initial OH, and this association remained significant after multivariable adjustment (OR 2.48, 95% CI 1.59–3.87) [10]. Longitudinal evidence also suggests that OH is associated with subsequent new-onset AF, with a pooled risk ratio of 1.48 (95% CI 1.21–1.81) in a meta-analysis of four cohort studies, although between-study heterogeneity was substantial (I^2^ = 69.4%) [11]. The available evidence therefore supports an association between AF and OH, but causality remains uncertain [10,11].

Taken together, these overlapping conditions can make BP assessment and treatment decisions particularly complex in older adults. In addition, frailty and multimorbidity may influence treatment tolerance and therapeutic priorities in this population [2,26].

## 4. Blood Pressure Assessment and Orthostatic Responses

In older adults with hypertension, AF, and OH, BP interpretation cannot rely on the average BP value alone. It also requires attention to measurement reproducibility, BP variability, and postural BP change [5,8,9].

### 4.1. Blood Pressure Measurement and Variability in Atrial Fibrillation

AF complicates BP measurement because its irregular rhythm causes pronounced beat-to-beat haemodynamic variation. Ventricular filling, stroke volume, and contractility vary from beat to beat, resulting in greater BP fluctuations than in sinus rhythm and reduced reproducibility of individual measurements [5]. Auscultatory measurement is also less reproducible in AF, with greater variation between observers and between repeated measurements by the same observer, particularly for diastolic BP. Greater intra-individual variability has also been reported with oscillometric measurements in AF than in sinus rhythm [5].

BP assessment in AF should therefore be based on repeated rather than isolated measurements. Stergiou et al. advise obtaining three readings during each measurement session, with additional assessment across separate office visits and outside the clinic [5]. The 2023 European Society of Hypertension (ESH) guidelines likewise recommend at least three office BP readings by auscultation in patients with AF and allow automated oscillometric measurement for office BP, while home and ambulatory BP assessment rely on automated oscillometric methods [27].

Automated oscillometric devices require additional caution in AF. Evidence from validation studies remains limited and heterogeneous, and performance varies between individual devices [5,14,28]. Stergiou et al.’s pooled analysis suggested generally acceptable systolic accuracy with a small upward bias in diastolic BP. Clark et al., however, found substantial heterogeneity and cautioned that performance could not be generalized from one device to another [5,28]. The presence of an AF-detection feature should not be taken as evidence that a device measures BP more accurately during AF [28]. ESH practice guidance also cautions that validation of a BP monitor in the general adult population does not establish its accuracy in patients with arrhythmias, particularly AF. Separate validation is therefore required in this population [29].

Available studies suggest that 24-h ambulatory blood pressure monitoring (ABPM) can be performed successfully in patients with stable AF, with a similar proportion of valid readings to that observed in sinus rhythm [5]. Taken together, these data support averaging repeated measurements and using devices with evidence of accuracy in patients with AF [5,28,29].

Beyond the beat-to-beat variability inherent to AF, BP also varies from day to day and between clinical visits. Because the arrhythmia itself contributes substantially to very-short-term BP fluctuations, longer time-scale measures, such as day-to-day or visit-to-visit variability, may be preferable for assessing BP variability in AF [6]. Higher visit-to-visit systolic BP variability has been associated with adverse outcomes in patients with AF. In a post hoc analysis of the AFFIRM study, greater visit-to-visit systolic BP variability was independently associated with stroke and major bleeding [30]. In a retrospective cohort of patients with AF and hypertension, thromboembolic risk was lower when tighter BP control was accompanied by less visit-to-visit variability [15]. Data from the J-RHYTHM Registry further support the prognostic relevance of visit-to-visit systolic BP variability for thromboembolism, major haemorrhage, and all-cause death [7]. However, BP variability estimates are method-dependent, universally accepted thresholds are lacking, and it remains uncertain whether reducing BP variability independently of mean BP improves clinical outcomes [31].

### 4.2. Orthostatic Blood Pressure Assessment

Orthostatic hypotension is conventionally defined as a sustained fall in systolic BP of ≥20 mmHg and/or diastolic BP of ≥10 mmHg within 3 min of standing [9,27]. The 2023 ESH guidelines recommend standing BP measurements at 1 and 3 min in adults aged >65 years and in other groups at increased risk of OH, including treated hypertensive patients, patients with diabetes or neurodegenerative disorders, and those with symptoms suggestive of postural hypotension [27]. The 2024 European Society of Cardiology (ESC) guidelines state that assessment for OH should be considered at the initial diagnosis of elevated BP or hypertension [26]. They also recommend testing before initiation or intensification of BP-lowering treatment, after 5 min in the sitting or lying position, with BP measured at 1 and/or 3 min after standing [26]. Because seated-to-standing assessment may underdetect OH, supine-to-standing testing is preferable when feasible, although seated-to-standing testing may be more practical in selected patients or clinical settings [8,9,26].

An orthostatic test should not be interpreted solely according to whether the diagnostic BP threshold is met. An asymptomatic result meeting the diagnostic criteria should not be dismissed solely because symptoms are absent [8,9]. A single positive result should nevertheless be interpreted in context, particularly in patients with hypertension, because within-person BP variability and measurement error can produce differences that cross the diagnostic threshold [9]. Conversely, a normal bedside test does not exclude OH when the clinical history is suggestive [8]. Because orthostatic symptoms may be nonspecific, the clinical history should specifically assess symptoms occurring on standing, including lightheadedness, presyncope, fatigue, visual blurring, difficulty concentrating, and neck or shoulder “coat-hanger” pain [32]. Smaller or transient postural BP reductions that do not meet the formal OH threshold may still be clinically relevant when they coincide with typical orthostatic symptoms [8,33]. Repeated measurements after relevant triggers, such as meals or exercise, or at different times of day may reveal abnormalities missed during a single bedside assessment [8,9]. BP self-measurement during symptoms or ambulatory BP monitoring accompanied by an activity diary may also be useful in this setting [8]. Delayed OH, which occurs after 3 min of standing, should be considered when typical postural symptoms persist despite a negative standard test [8,9]. If the clinical assessment remains inconclusive, more specialized evaluation may be appropriate. Tilt-table testing can be useful, particularly for delayed OH; continuous beat-to-beat BP measurement during active standing can identify initial or transient BP falls; and autonomic function testing may help clarify the underlying mechanism [8].

In older adults with hypertension, orthostatic BP changes should also be considered in relation to supine and nocturnal BP. Supine hypertension can coexist with OH and, in patients with neurogenic OH, may reflect hypertension that predates autonomic disease or emerge as a consequence of autonomic failure itself [9]. Supine BP should therefore be assessed, particularly when neurogenic OH is suspected [8]. Nocturnal hypertension may promote pressure natriuresis and overnight volume loss, contributing to worse orthostatic tolerance in the morning, particularly in patients with autonomic failure [9]. ABPM can characterize nighttime BP directly, including the presence of nocturnal hypertension and the nocturnal dipping pattern [26]. Orthostatic BP findings should therefore be interpreted within the broader BP profile rather than in isolation.

In patients with AF, interpretation of the heart-rate response during orthostatic testing requires caution. A disproportionately small increase in heart rate relative to the fall in systolic BP can support a neurogenic mechanism of OH, but this relationship cannot be reliably applied in patients with AF or in those taking β-blockers because both alter the heart-rate response [8]. The heart-rate response should therefore be interpreted cautiously in the context of the broader clinical assessment and should not be used alone to diagnose neurogenic OH.

## 5. Treatment-Related Factors Affecting Blood Pressure and Orthostatic Tolerance

Detection of OH in an older patient with hypertension should prompt reassessment of treatment, but not automatic deintensification. The 2023 ESH guidelines recommend individualized BP targets in frail patients and state that treatment reduction may be considered in patients aged ≥80 years with low systolic BP (<120 mmHg), severe OH, or a high level of frailty [27]. The 2024 ESC guidelines recommend the usual BP-lowering approach in older adults aged <85 years who are not moderately to severely frail when treatment is well tolerated [26]. More individualized BP targets may be considered in patients with pre-treatment symptomatic OH, those aged ≥85 years, or those with moderate-to-severe frailty [26]. As frailty progresses, systolic BP may fall, and deprescribing may become appropriate [26]. In patients with supine hypertension and OH, ESC guidance specifically recommends replacing BP-lowering drugs that worsen OH rather than simply reducing treatment intensity [26]. The 2025 American Heart Association/American College of Cardiology multisociety guideline similarly recommends improved BP control in adults with hypertension and considers assessment for symptomatic OH reasonable when treatment is initiated or an additional antihypertensive medication is added [34].

Randomized trial evidence also argues against using baseline OH alone as a reason to avoid more intensive BP lowering. A systematic review and individual participant data meta-analysis of five blood-pressure-target trials found that more intensive BP treatment was associated with slightly lower odds of OH (OR 0.93, 95% CI 0.86–0.99) [16]. In a meta-analysis of individual-level data from 29,235 adults in nine hypertension trials, assignment to more intensive or active BP-lowering treatment reduced the risk of cardiovascular disease or all-cause mortality to a similar extent in those with and without baseline OH [17]. These findings should not, however, be extended uncritically to all older patients. Trial selection may have limited representation of patients with low standing BP, neurogenic or more severe OH, and frailty, while dizziness, falls, and syncope were not systematically captured in many studies. Orthostatic assessment was based on seated-to-standing measurements, and many protocols neither covered the full 3-min period nor confirmed OH on repeat testing. Generalizability to frail older adults with symptomatic, severe, or neurogenic OH therefore remains uncertain [17].

Evidence on withdrawal of antihypertensive therapy is less straightforward. In the DANTE trial, which included community-dwelling adults aged ≥75 years with mild cognitive impairment and OH, discontinuation of antihypertensive treatment did not significantly increase resolution of OH in the intention-to-treat analysis. Per-protocol analyses suggested a higher probability of recovery from OH among those who actually discontinued treatment, particularly among those who discontinued all antihypertensive medication, but this finding does not establish a general benefit of treatment withdrawal [18]. A 2024 systematic review further highlighted the limited certainty of the available evidence. OH prevalence decreased after initiation or intensification of antihypertensive treatment, but also after discontinuation. The latter estimate was based on only two studies involving 140 individuals. Substantial differences in antihypertensive classes, study designs, and methods of OH assessment, together with very low certainty of evidence, limit firm conclusions [19]. Taken together, these findings do not support a simple rule either to intensify or to withdraw antihypertensive treatment whenever OH is detected.

Drug class and clinical indication therefore matter. In a systematic review and meta-analysis of 69 placebo-controlled randomized trials, β-blockers were associated with markedly higher odds of OH (OR 7.76, 95% CI 2.51–24.03). A more modest association was observed with α-blockers (OR 1.87, 95% CI 1.33–2.64). No significant association was identified for calcium-channel blockers or angiotensin-converting enzyme (ACE) inhibitors/angiotensin receptor blockers (ARBs) versus placebo [20]. The applicability of these findings to older adults is limited, as the mean age was 56.1 years in the β-blocker studies and 55.9 years in the α-blocker studies. In addition, the meta-analysis did not include head-to-head comparisons between drug classes [20]. Among ALLHAT participants aged ≥65 years, long-term risks of OH, syncope, and falls did not differ significantly between chlorthalidone, amlodipine, and lisinopril. Amlodipine was, however, associated with a higher risk of falls during the first year [21]. Different drugs can compromise orthostatic adaptation through different mechanisms. Some attenuate vasoconstrictor or chronotropic/inotropic compensation, some promote venous pooling, and others reduce intravascular volume [35].

The distinction between drug effect and drug indication is particularly important in AF. β-Blockers may impair compensatory responses to standing, yet they may still be required for ventricular rate control. Current ESC guidance recognizes heart-rate control as a compelling indication for β-blocker therapy [26,35]. In frail older patients, age- and frailty-related changes in pharmacokinetics and pharmacodynamics may increase susceptibility to bradycardia and other adverse effects of rate-slowing therapy [12]. In patients with coexisting hypertension, AF, and OH, medication review should therefore extend beyond antihypertensive drugs and consider the complete regimen. Drugs that may worsen orthostatic BP should be reviewed in relation to their indication and expected benefit, with dose reduction, discontinuation, or substitution considered when appropriate. Redundant agents are particularly suitable for deprescribing or replacement with better-tolerated alternatives [25,35,36]. In frail patients, functional status, life expectancy, treatment priorities, and tolerance should also inform these decisions [25,37]. More broadly, medication review in older adults with AF and multimorbidity should take into account overall health, functional capacity, and the patient’s individual priorities when treatment choices are made [38]. When BP-lowering therapy is initiated or changed, low starting doses, gradual titration, and subsequent assessment for OH and related symptoms are appropriate [25]. Non-pharmacological measures may complement medication review in patients with OH, but their selection should be individualized because feasibility and haemodynamic responses vary among older adults [39,40]. Treatment review should therefore focus not simply on whether OH is present, but on which therapy may be contributing to orthostatic intolerance, whether its indication remains compelling, and whether the same therapeutic goal can be achieved with better tolerance.

## 6. Frailty and Falls as Modifiers of Treatment Decisions

Frailty becomes increasingly common with advancing age and reflects diminished physiological resilience, which may reduce tolerance of BP-lowering and rate-control therapies and increase vulnerability to treatment-related adverse events and other stressors [12,37]. Its prevalence is particularly high in patients with AF [41,42]. In a systematic review and meta-analysis of 33 studies including 1,187,651 patients with AF, the pooled prevalence of frailty was 39.7% (95% CI 29.9–50.5%). This estimate varied markedly across studies (I^2^ = 100%), with differences related to factors including the method used to assess frailty and the clinical setting [41]. Frailty was also associated with higher odds of all-cause death, ischaemic stroke, and bleeding, although heterogeneity was high for these comparisons [41].

Falls are an important component of this clinical vulnerability. In a systematic review and meta-analysis of 184 studies, pooled unadjusted ORs for falls during a 12-month reporting interval were 1.52 (95% CI 1.27–1.82) for AF and 1.39 (95% CI 1.18–1.64) for OH [22]. In a large English primary-care cohort of adults aged ≥65 years with AF who were eligible for oral anticoagulation, increasing frailty was associated with a higher risk of falls. Patients with severe frailty had an adjusted hazard ratio of 8.03 (95% CI 4.60–14.03) compared with fit patients [42]. The causes of falls in frail older adults are often multiple, with potential contributors including OH or low BP, medication effects, gait and balance impairment, cognitive impairment, and arrhythmias [12,43]. Thus, the presence of OH does not by itself establish the mechanism of a fall, particularly in an older patient with AF in whom several cardiovascular and non-cardiovascular contributors may coexist [12,43]. These factors should therefore be considered together when treatment is reviewed after a fall [12,43].

A history of falls also identifies patients at increased risk of subsequent adverse events. In a post hoc analysis of the prospective ANAFIE Registry of Japanese patients aged ≥75 years with non-valvular AF, previous falls were independently associated with stroke or systemic embolism, major bleeding, intracranial haemorrhage, all-cause mortality, and subsequent falls or fractures [44]. These associations should be interpreted cautiously. Detailed information on frailty and activities of daily living was not available, so fall history may partly have captured underlying frailty that was not measured directly, and residual confounding by frailty cannot be excluded [44].

For patients receiving anticoagulation, falls raise particular concern about traumatic bleeding, especially intracranial haemorrhage. Fall risk alone, however, does not generally justify withholding oral anticoagulation when stroke prevention is otherwise indicated. The European Heart Rhythm Association (EHRA) Practical Guide does not regard falling alone as a contraindication to direct oral anticoagulant (DOAC) therapy [13]. It recommends addressing modifiable bleeding risks and implementing measures to reduce further falls [13]. The EHRA consensus on arrhythmias in frailty similarly states that frailty, cognitive decline, or fall risk should not generally preclude anticoagulation when otherwise indicated [12]. Decisions should consider stroke and bleeding risk as well as patient preferences [12]. Cognitive impairment may nevertheless complicate medication self-management, particularly in patients receiving multiple therapies, and may increase the need for caregiver support in organizing and monitoring treatment [45,46]. This is particularly relevant in AF because non-adherence to oral anticoagulation may affect up to 30% of patients, and lower adherence has been associated with stroke and mortality [47]. Current ESC guidelines emphasize correction of modifiable bleeding risks, including strict BP control in patients with hypertension, avoidance of unnecessary antiplatelet or anti-inflammatory drugs, and review of concomitant medications for relevant interactions [1]. In an older patient with AF, OH, and recurrent falls, management should therefore aim to reduce reversible contributors to falls and bleeding without withholding anticoagulation solely because falls have occurred.

The choice of anticoagulant requires additional consideration in frail older adults. Current ESC guidelines recommend DOACs in preference to vitamin K antagonists (VKAs) in eligible patients with AF, except those with mechanical heart valves or moderate-to-severe mitral stenosis [1]. However, this general preference does not imply that every clinically stable older patient receiving a VKA should be switched. In the FRAIL-AF randomized trial, patients aged ≥75 years with AF and frailty who were receiving international normalized ratio (INR)-guided VKA treatment were randomized either to continue VKA treatment or to switch to a DOAC. Switching was associated with a higher incidence of major or clinically relevant non-major bleeding (hazard ratio 1.69, 95% CI 1.23–2.32), without a reduction in thromboembolic events [23]. FRAIL-AF addressed a specific clinical question—the effect of switching patients already established on VKA therapy—and should not be interpreted as evidence that DOACs are generally inferior in frail patients. The choice of individual DOAC was not randomized, and the trial does not allow conclusions for patients with poor INR control, for whom switching may remain appropriate [23]. Consistent with these findings, ESC guidance allows continuation of clinically stable therapeutic VKA treatment in patients aged ≥75 years with polypharmacy rather than routine switching to a DOAC [1]. Frailty and fall risk should therefore influence how anticoagulation is selected, monitored, and reviewed, rather than serve as automatic reasons either to withhold stroke-prevention therapy or to alter an otherwise stable anticoagulant regimen.

## 7. Discussion and Future Perspectives

**Figure 1:**
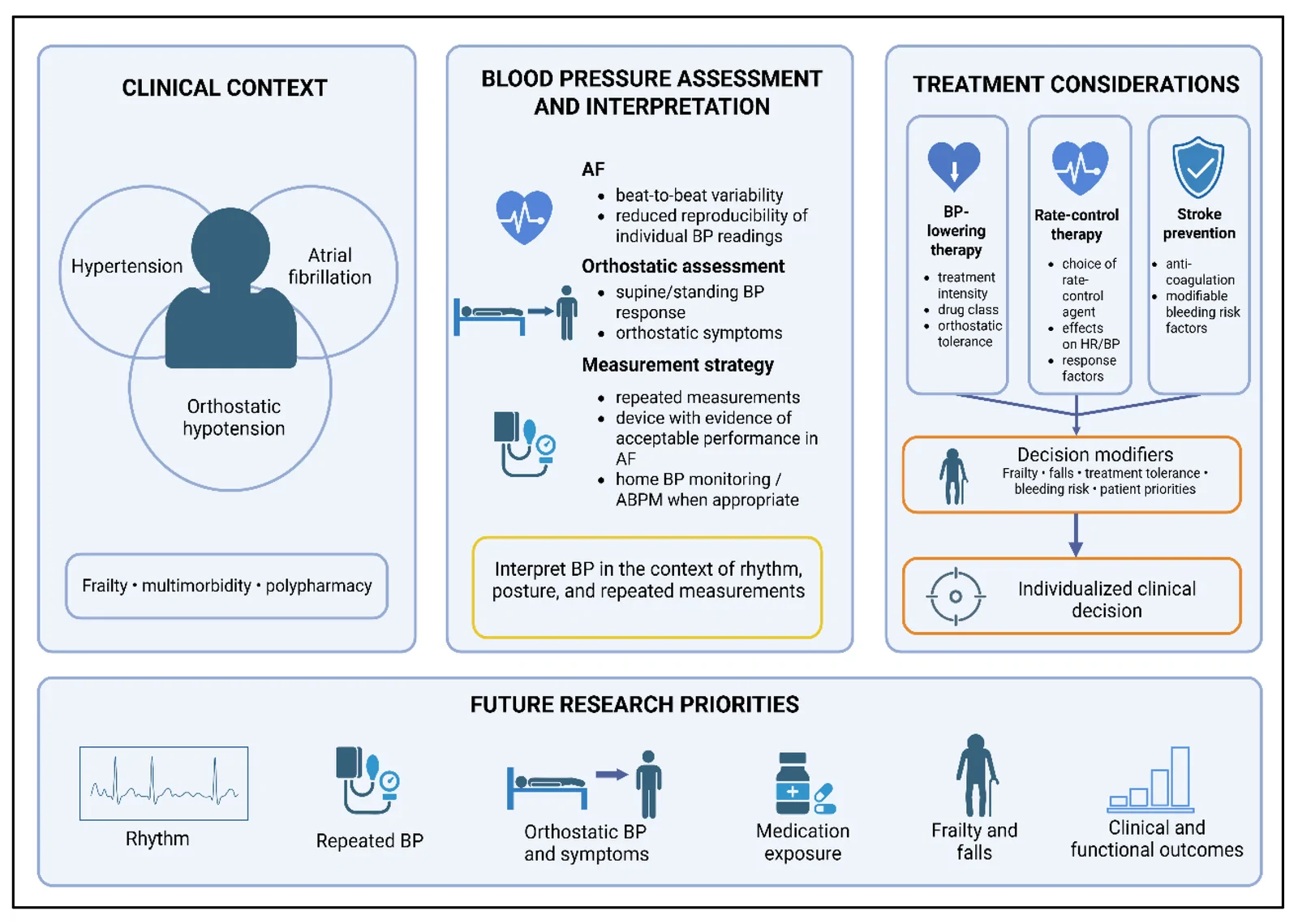
Blood pressure assessment and treatment considerations in older adults with coexisting hypertension, atrial fibrillation, and orthostatic hypotension. The figure integrates the clinical context, blood pressure assessment and interpretation, treatment considerations, decision modifiers, and priorities for future research. Reliable blood pressure assessment provides the basis for individualized treatment decisions that account for rhythm, orthostatic responses, frailty, falls, treatment tolerance, bleeding risk, and patient priorities. Abbreviations: AF, atrial fibrillation; BP, blood pressure; ABPM, ambulatory blood pressure monitoring; HR, heart rate. Created in BioRender. Kabaklic, A. (2026) https://BioRender.com/xygnfpv (accessed on 20 September 2026).

The available evidence suggests that treatment decisions in older adults with hypertension, AF, and OH should be based on an integrated assessment of BP measurement reliability, orthostatic responses, symptoms, treatment tolerance, and the overall clinical context. In AF, repeated BP measurements are preferable to isolated readings because beat-to-beat variability may reduce measurement reproducibility [5]. Device performance also requires attention, because accuracy of BP measurement during AF and accuracy of automated AF detection are distinct issues and should not be conflated [28,48]. Detection of OH should not automatically prompt deintensification of antihypertensive therapy, as randomized evidence suggests that baseline OH does not eliminate the cardiovascular benefit of BP lowering [17]. However, the available evidence is less directly applicable to patients with symptomatic or severe OH, low standing BP, frailty, or recurrent falls, who remain underrepresented in clinical trials [17,19]. Overall, these findings support treatment decisions based on the overall BP profile, symptoms, treatment tolerance, medication indications, frailty, and fall risk rather than on any single BP value or on the presence of OH alone [17,19]. Frailty and falls should prompt careful treatment review, including anticoagulation decisions, but should not by themselves justify withholding otherwise indicated anticoagulation [12,13]. In frail older patients who are stable on VKA therapy, randomized evidence also argues against routine switching to a DOAC without a clear indication [23]. The main clinical considerations and future research priorities are summarized in Figure 1.

This review has limitations. Its focused narrative design did not include a formal risk-of-bias assessment or quantitative synthesis. PubMed was the only bibliographic database searched, and several of the focused search strategies included Title restrictions. In addition, only English-language human publications were considered during screening. Consequently, relevant studies indexed exclusively in other databases, not captured by the focused search strategies, or published in languages other than English may have been missed, although reference-list screening of key reviews, guidelines, and original studies was used to broaden literature identification. More importantly, the evidence considered here was derived from overlapping, but not identical populations, and few studies examined measurement, treatment, and geriatric factors together. Orthostatic measurement protocols, frailty assessments, and treatment exposures differed substantially across studies, while frail and multimorbid older adults were not consistently represented in randomized trials [2,17,19,41]. The clinical implications presented here should therefore be regarded as a synthesis of heterogeneous evidence rather than as support for a single management strategy.

The most important remaining evidence gap concerns BP assessment itself when AF and OH coexist. Even within the better studied relationship between hypertension and AF, optimal BP targets for AF-related outcomes and the preferred antihypertensive strategy remain uncertain [3]. Evidence on hypertension and OH is limited by inconsistent orthostatic assessment [19], while evidence relevant to frail older adults with AF remains limited, particularly for stroke-prevention decisions and for the clinical implications of falls, orthostatic intolerance, and treatment-related adverse effects [2,12]. Prospective studies are needed to determine how BP and hypertension should be assessed when AF-related measurement variability and orthostatic BP changes are present in the same older patient, and how this assessment should guide treatment. Such studies should combine repeated BP and rhythm assessment with standardized supine and standing BP measurements, orthostatic symptoms, medication exposure, frailty, falls, anticoagulation, and cardiovascular, bleeding, functional, and quality-of-life outcomes. This would allow both the reliability of BP assessment and its consequences for treatment decisions to be studied directly, rather than inferred across separate evidence bases.

## 8. Conclusions

Hypertension, AF, and OH are common in older adults, and their coexistence complicates both BP assessment and treatment decisions. In patients with AF, BP assessment should rely on repeated rather than isolated measurements, while orthostatic assessment provides information that cannot be inferred from seated BP alone. A positive orthostatic test should not be dismissed because symptoms are absent, but neither should the detection of OH automatically lead to deintensification of antihypertensive therapy. Treatment decisions should consider the broader BP profile, treatment tolerance, and whether drugs that may impair orthostatic compensation remain necessary for another cardiovascular indication, particularly rate control in AF. Frailty and falls further influence treatment tolerance and bleeding risk. However, fall risk alone should not lead to withholding indicated anticoagulation or to routine alteration of an otherwise stable anticoagulant regimen.

Management of older adults with overlapping cardiovascular and geriatric conditions necessarily requires an individualized and integrated approach. Individualization should not replace standardized assessment but should be built on it. Treatment decisions should be based on reliable BP measurement, validated measurement protocols and devices, and the best available evidence and guideline recommendations. This approach can then be adapted to the patient’s multimorbidity, frailty, symptoms, treatment tolerance, and priorities. A major remaining evidence gap is how BP and hypertension should be reliably assessed when AF and OH coexist in the same older patient, and how this assessment should guide treatment decisions.

## Figures and Tables

**Table 1:** Key evidence informing BP assessment and treatment decisions in older adults with hypertension, AF, and OH.

Study; Design	Population and Age	BP/OH Assessment	Treatment/Exposure	Main Outcomes and Findings	Main Limitations and Applicability
Stergiou et al., 2019 [5]; systematic review/meta-analysis	AF; 14 articles including 12 non-invasive validation studies (*n* = 781) and 4 invasive comparisons (*n* = 179); age varied	Office, home, and ambulatory BP; auscultatory, automated, and intra-arterial methods; OH not assessed	BP measurement method/device in AF	AF increases measurement variability; triplicate readings are recommended. Automated devices show reasonable SBP accuracy with small DBP overestimation; 24-h ambulatory BP monitoring is feasible.	Validation evidence was limited and methodologically heterogeneous. Directly applicable to BP measurement in AF, not to OH assessment.
Mai et al., 2022 [14]; observational comparison study	*n* = 202 with AF; mean age 73.5 y; 62.4% had hypertension	10-min seated rest; 2 readings averaged; simultaneous mercury, pulse-wave, and oscillographic measurements	BP measurement method/device	Pulse-wave measurements showed closer agreement with mercury sphygmomanometry than oscillographic measurements.	Single-center inpatient cohort; mercury rather than invasive reference; only 2 readings; heart rates were mostly normal. Relevant to older adults with AF.
Kim et al., 2022 [15]; retrospective cohort	*n* = 13,532 with AF; *n* = 9505 with hypertension; mean age 66.7 y in the hypertensive group	5-min seated rest; automated office BP; ≥3 readings/visit, last 2 averaged; serial follow-up measurements	BP level, visit-to-visit variability, and BP time in therapeutic range	Lower visit-to-visit BP variability and higher SBP time in therapeutic range were associated with lower ischemic stroke/systemic embolism risk; patients with SBP time in therapeutic range >94% (<130 mmHg) had risk comparable to those without hypertension.	Retrospective single-center registry; selection/generalizability concerns, variable follow-up, and relatively low anticoagulant use; OH not assessed.
McNicholas et al., 2017 [10]; cross-sectional analysis of Wave 1 of the prospective TILDA cohort	*n* = 4408 community-dwelling adults aged ≥50 y; *n* = 101 with AF	Beat-to-beat BP during 110 s active stand; sustained classical OH ≥20/10 mmHg, assessed at 30, 60, and 90 s; initial OH ≥40/20 mmHg at 10 s	Presence of AF	AF was independently associated with initial OH (OR 2.48) and OH at 30 s (OR 1.78).	Cross-sectional analysis; low AF prevalence; paroxysmal AF may have been missed; comorbidities were self-reported. Supports association, not causality.
Juraschek et al., 2021 [16]; systematic review/IPD meta-analysis	5 primary BP-target trials, *n* = 18,466 (mean age 64.5 y; *n* = 2895 aged >75 y); 4 placebo-controlled trials in sensitivity analyses	OH ≥20/10 mmHg; seated-to-standing; timing and frequency varied across trials	Intensive vs. standard BP targets	More intensive BP treatment lowered the odds of OH (OR 0.93, 95% CI 0.86–0.99).	First-minute OH was incompletely captured; falls/syncope data were unavailable; strict trial criteria limit applicability to frail adults.
Juraschek et al., 2023 [17]; IPD meta-analysis of randomized trials	*n* = 29,235 from 9 hypertension trials; mean age 69.0 y; 9% had baseline OH	OH ≥20/10 mmHg; seated-to-standing; protocols varied across trials	More intensive BP target/active therapy vs. standard target/placebo	More intensive BP treatment reduced the risk of the composite of CVD or all-cause mortality similarly in participants without baseline OH (HR 0.81, 95% CI 0.76–0.86) and with baseline OH (HR 0.83, 95% CI 0.70–1.00; interaction P = 0.68).	Low standing BP, neurogenic/severe OH, and frailty were underrepresented; participants with OH were not necessarily symptomatic; falls/syncope were not assessed in many trials.
Moonen et al., 2016 [18]; randomized controlled trial	*n* = 162 with baseline and 4-month OH data; age ≥75 y; median age 80.4 y (discontinuation) and 81.6 y (continuation); mild cognitive impairment, OH, and antihypertensive use	5-min seated rest; 2 seated then 3 standing readings within ≈3 min; OH ≥20/10 mmHg	Antihypertensive discontinuation vs. continuation	In the intention-to-treat analysis, OH resolution was not significantly higher with antihypertensive discontinuation (RR 1.31, 95% CI 0.92–1.87). In the per-protocol analysis, complete discontinuation was associated with greater OH resolution (RR 1.60, 95% CI 1.10–2.31).	Strict exclusion criteria limit generalizability to older adults without dementia or serious CVD; power may have been insufficient for the ITT comparison; seated-to-standing measurements may have caused OH misclassification.
Klop et al., 2024 [19]; systematic review/meta-analysis	25 studies; *n* = 26,695 hypertensive adults aged ≥65 y; 15 studies contributed to meta-analyses	OH definitions, posture, timing, and measurement methods varied substantially	Initiation, augmentation, or discontinuation of antihypertensive treatment	OH prevalence decreased after initiation or augmentation of antihypertensive treatment (RR 0.39, 95% CI 0.21–0.72; 6 studies) and after discontinuation (RR 0.39, 95% CI 0.28–0.55; 2 studies); postural BP drop was unchanged after initiation or augmentation.	OH methods were heterogeneous; 20 studies had moderate-to-serious risk of bias; overall evidence was low, and few studies included frail older adults.
Bhanu et al., 2021 [20]; systematic review/meta-analysis	69 placebo-controlled randomized trials; 27,079 participants; mean age varied by drug class, and older adults were underrepresented	OH definitions and ascertainment varied across trials	Drug classes versus placebo, including β-blockers, α-blockers, calcium-channel blockers, and ACE inhibitors/ARBs	β-blockers were associated with higher odds of OH (OR 7.76, 95% CI 2.51–24.03), as were α-blockers (OR 1.87, 95% CI 1.33–2.64). No significant association was found for calcium-channel blockers or ACE inhibitors/ARBs versus placebo.	No head-to-head comparisons between drug classes; limited applicability to older adults because participants in several drug-class analyses were relatively young.
Juraschek et al., 2019 [21]; secondary analysis of ALLHAT	23,964 hypertensive adults aged ≥65 years; mean age 69.8 years	Falls, syncope, and OH identified from administrative claims; no standardized orthostatic BP protocol.	Randomized first-step treatment with chlorthalidone, amlodipine, or lisinopril.	Long-term risks of falls, syncope, and OH did not differ significantly between treatment groups. During the first year, amlodipine was associated with a higher risk of falls than chlorthalidone (HR 2.24, 95% CI 1.06–4.74) and lisinopril (HR 2.61, 95% CI 1.03–6.72).	Outcomes were based on administrative claims rather than standardized prospective OH assessment; directly relevant to older adults with hypertension.
Bourke et al., 2024 [22]; systematic review/meta-analysis	184 studies in adults aged ≥50 y (181 observational, 3 interventional)	67 observational studies assessed OH and falls; OH definitions/assessment methods varied (beat-to-beat, oscillometric, auscultatory, or unspecified)	Cardiovascular disorders, including AF and OH, in relation to falls	In 12-month unadjusted meta-analyses, AF (6 studies) was associated with falls (OR 1.52, 95% CI 1.27–1.82) and OH (25 studies) with falls (OR 1.39, 95% CI 1.18–1.64).	Predominantly observational evidence; pooled ORs were unadjusted; wide variation in sampling frames, study designs, assessment methods, and quality-assessment scores. Causal inferences are limited.
Joosten et al., 2024 [23]; pragmatic multicenter randomized trial	*n* = 1323 in the ITT population; AF, age ≥75 y and Groningen Frailty Indicator ≥3; mean age 83 y	BP/OH was not assessed as part of the study question	Switch from INR-guided VKA to DOAC vs. continued VKA	Switching increased major/clinically relevant non-major bleeding (HR 1.69, 95% CI 1.23–2.32) without reducing thromboembolic events.	Open-label; DOAC choice was nonrandomized; individual TTR was not recorded. Most applicable to patients receiving adequate INR-guided VKA; the trial does not allow conclusions for low-TTR patients and did not address VKA-naïve patients.

Abbreviations: ACE, angiotensin-converting enzyme; AF, atrial fibrillation; ARB, angiotensin receptor blocker; BP, blood pressure; CI, confidence interval; CVD, cardiovascular disease; DBP, diastolic blood pressure; DOAC, direct oral anticoagulant; HR, hazard ratio; INR, international normalized ratio; IPD, individual participant data; ITT, intention-to-treat; OH, orthostatic hypotension; OR, odds ratio; RR, risk ratio; SBP, systolic blood pressure; TTR, time in therapeutic range; VKA, vitamin K antagonist. Note: Because evidence in older adults with coexisting hypertension, AF, and OH was scarce, partially overlapping populations were included when directly relevant. OH definitions and measurement protocols are reported as used and are not assumed to be equivalent.

## Data Availability

No new data were created or analyzed in this study. Data sharing is not applicable to this article.

## Abbreviations

The following abbreviations are used in this manuscript:

AF	atrial fibrillation
ABPM	ambulatory blood pressure monitoring
ACE	angiotensin-converting enzyme
ARB	angiotensin receptor blocker
BP	blood pressure
CI	confidence interval
DOAC	direct oral anticoagulant
EHRA	European Heart Rhythm Association
ESC	European Society of Cardiology
ESH	European Society of Hypertension
INR	international normalized ratio
OH	orthostatic hypotension
OR	odds ratio
VKA	vitamin K antagonist
